# Ruminal Lipopolysaccharides Analysis: Uncharted Waters with Promising Signs

**DOI:** 10.3390/ani11010195

**Published:** 2021-01-15

**Authors:** Efstathios Sarmikasoglou, Antonio P. Faciola

**Affiliations:** Department of Animal Sciences, University of Florida, Gainesville, FL 32611, USA; sarmikasoglou.ef@ufl.edu

**Keywords:** extraction protocol, LBP, *Prevotella*, single molecule localization microscopy, ruminal LPS

## Abstract

**Simple Summary:**

Lipopolysaccharide (LPS) is a component of the outer membranes of Gram-negative bacterial cell wall made of three covalently linked regions: the O-antigen, the core oligosaccharide, and the endotoxin lipid A moiety, which carries the endotoxic activity of LPS. Among Gram-negative bacteria there is significant structural diversity in the lipid A region. Specifically, the number of lipid A acyl chains directly correlates with the ability to induce cytokine production whereas the hexa-acylated forms usually are the most immunostimulant ones, contrary to penta- or tetra- acylated forms that result in weak inflammatory host responses. Ruminal bacteria are predominantly Gram-negative, and their respective LPS presence has been suggested to be associated with ruminal acidosis, a metabolic disorder of cattle with negative effects on health and production. In the rumen, the most predominant phylum is *Bacteroidetes* which exhibit weak host immunological response compared to widely used *Escherichia coli* LPS. This review aims to present accumulated knowledge regarding ruminal LPS, pointing out the differences in ruminal LPS compared to widely known LPS, and introduce hypotheses that could contribute to further understanding and planning strategies to tackle ruminal acidosis.

**Abstract:**

The objective of this review is to present the need for the development of a comprehensive ruminal lipopolysaccharide (LPS) extraction, purification and analysis protocol and state hypotheses that could contribute to planning novel strategies against ruminal acidosis. Lipopolysaccharide is an immunostimulatory molecule of Gram-negative bacterial outer membranes and has been reported to contribute to ruminal acidosis in cattle. Bacterial death and lysis are normal processes, and thus LPS is normally present in ruminal fluid. However, ruminal LPS concentration is much greater during subacute ruminal acidosis (SARA). Contrary to the widely known LPSs, ruminal LPS seems to be composed of a variety of LPS chemotypes that may interact with each other resulting in an LPS “mixture”. Hypotheses regarding the influence of each specific ruminal bacterial specie to innate immunity during SARA, and the representativeness of the exclusive use of the *Escherichia coli* LPS to rumen epithelial tissue challenges, could expand our knowledge regarding SARA. In addition, possible correlation between the monomeric Toll-like Receptor 4 (TRL4) and the antagonistic penta-acylated lipid A of LPS could contribute to novel strategies to tackle this nutrition disorder.

## 1. Introduction

Lipopolysaccharides (LPS) are surface molecules of Gram-negative bacterial cell wall. In the early 1900s much of the attention LPS received was due to its immunostimulatory ability [1]. However, it was later discovered that LPS protects the Gram-negative bacteria against bile salts and lipophilic antibiotics, thus, contributing to conservation of bacterial structural and functional integrity [2,3]. These two important functions have led many researchers to pursue further investigations into the physiology and biogenesis of LPS mostly using *Escherichia coli* and *Salmonella spp*. as model systems [4]. Elevated levels of LPS in blood plasma are associated with multiple diseases including ulcerative colitis [5], Crohn’s disease [6], autism [7], Alzheimer’s disease [8], and obesity in humans [9], as well as ruminal acidosis in bovines [10]. One of the strategies most commonly used by researchers and producers worldwide is the use of feed additives to improve animal health and production during the post weaning period [11,12]. However, some synthetic feed additives are prohibited by the European Union [13] and the United States [14], because of the potential anti-microbial resistance and possible impact on human health. These regulations open a wide niche for researchers to evaluate alternative products such as probiotics, prebiotics, enzymes, organic acids, organic minerals, plant extracts, and essential oils, as well as modifications of LPS that could have a positive impact on health and production of animals. More specifically, a widely used strategy that has applications in vaccine development is the modification of bacterial strains to produce alternative LPS glyco-forms by overexpressing and/or knocking out genes involved in LPS biosynthesis [15,16]. By extension, these modifications can result into a range of cytokine response [15].

In cattle, feeding diets high in grain are a common practice; however, when the consumption of rapidly fermentable carbohydrates increases, so can the excessive accumulation of acids in the rumen, resulting in ruminal acidosis [10]. Ruminal acidosis is a metabolic disorder which has been associated with reduced feed intake, milk production, and milk fat depression [17,18]. The classification of ruminal acidosis as acute or subacute (SARA), is based on ruminal pH, total organic acid concentration, and evident clinical signs [19]. Acute cases are primarily characterized by the presence of clinical signs, ruminal pH below 5, and mortality [10], thus may be easily diagnosed; on the other hand, subacute cases exhibit no clinical signs and episodes of ruminal pH between 5.0 to 5.5 for more than 180min/d [20], and therefore are difficult to be identified. In cattle with SARA, concentrations of ruminal LPS and blood plasma LPS are, on average, greater than cows without SARA [21,22]. LPS also affects ruminal fermentation and bacteria diversity by stimulating the Gram-negative bacteria associated with starch digestion [23]. Additionally, LPS can be used as a substrate for acidosis related bacteria including *Streptococcus bovis* and *Selenomonas ruminantium* [24]; thus, LPS has long been suspected of contributing to the pathogenesis of SARA [25].

Ruminal bacteria are predominantly Gram-negative and are the major source of LPS in the rumen [26]. The presence of LPS in the ruminal fluid is normal since bacterial death and lysis are normal processes that take place during ruminal fermentation; however, under SARA conditions, LPS concentration is much greater compared to healthy cattle [19,27]. Contrary to the widely known LPS (e.g., *E. coli* LPS), ruminal LPS seems to be composed of a variety of LPS chemotypes that may interact with each other resulting in an LPS “mixture”, meaning that there is likely to be a broad spectrum of ruminal-Gram-negative bacteria contributing in different amounts to that mixture in each instance of pathogenic and healthy state, in order to compose the actual ruminal LPS.

The influence of each specific ruminal bacterial specie on innate immunity during SARA, the representativeness of exclusively using *Escherichia coli* LPS to investigate inflammation in cattle, and the possible correlation between the monomeric Toll-like Receptor 4, a cell receptor for LPS, and the antagonistic penta-acylated LPS from ruminal *Bacteroidetes*, are unknown. These factors could expand our knowledge regarding SARA and contribute to novel strategies to tackle this metabolic disorder. However, these issues, cannot currently be well investigated because of the lack of a well-established protocol for the complete extraction, purification and analysis of ruminal LPS, adjusted to specificities of ruminal Gram-negative bacteria. To our knowledge of the published literature, this is the first review exhibiting accumulated knowledge regarding ruminal LPS, pointing out the differences in ruminal LPS compared to widely known *E. coli* LPS, highlighting the importance of developing a comprehensive extraction, purification and analysis of ruminal LPS protocol adapted to the peculiarities of ruminal bacteria, and pointing out its contribution towards answering fundamental hypotheses about the understanding of SARA.

## 2. Lipopolysaccharides Extraction Protocols

Lipopolysaccharide molecule bioactivity varies among different bacteria, because of their different structures and amphipathic properties, and there is no panacea method for extraction of every LPS variety. Structurally, LPS comprises three covalently linked regions: the lipid A (endotoxin), the rough core oligosaccharide, and the O-antigenic side chain, determining serotype specificity [28]. Wild-type LPS contains the O-antigenic side chain and is referred to as smooth. Rough LPS is less common and does not contain the O-side chain [29]. The LPS extraction methods developed thus far favor specific serovars and phenotypes of Gram-negative bacteria. For instance, Galanos et al., 1969 [30], described an ether extraction method that favors the extraction of LPS from bacteria that express rough phenotypes. In contrast, Hickman and Ashwell, [31] proposed the phenol-water method that favors LPS extraction from bacteria that express smooth phenotypes. Apart from the presence or absence of O-antigen (smooth or rough), the extraction methods differ depending on the bacterial species being used to propagate LPS. More specifically, a hot-phenol extraction method was used for LPS isolation from *Burkholderia pseudomallei* [32], *Salmonella typhi* [33] as well as other well-known bacteria species; however, each specie exhibits variations in LPS molecular weight, as well as resistance to reagents used by different protocols, thus modifications are required in respective extraction protocols.

In general, various methods have been developed and modified by researchers in order to fit their needs. These include extraction with pyridine [34], trichloroacetic acid [35], phenol [36], water [37], ether [30] butanol [38], and sodium dodecyl sulfate (SDS); [39]. Among these methods the phenol extraction is indicated for smooth LPS extraction while ether extraction is more efficient for rough LPS extraction.

### Ruminal Lipopolysaccharides Extraction, Purification, and Quantification

The extraction protocol developed by Westphal and Jann [36] was used by Berczi et al. [40], to extract the endotoxin from *E. coli* O78 LPS in order to measure the sensitivity of different species, including calves and swine. Later, Nagaraja et al. [41] investigated the endotoxic activity of cell-free rumen fluid from cattle by pursuing the first documented ruminal LPS extraction based on the phenol-water method [36] purified with ultracentrifugation and cetyltrimethylammonium bromide (CTAB), as well as the aqueous ether [35] method. Despite the addition of the CTAB purification procedure by Nagaraja being novel, the quality of the preparation cannot be validated because neither UV spectrophotometric analysis nor staining methods were used. In addition, Hitchcock and Brown [42] studied the morphological heterogenicity among *Salmonella* LPS chemotypes and proposed further purification processes to the Westphal and Jann protocol to mitigate the contamination from proteins, especially lipoproteins. There are no recent documented attempts at ruminal LPS extraction and purification; however, research in animal science is centered on quantification. A widely used technique is the centrifugation of strained rumen fluid for 30 min at 10,000× *g*, passing the supernatant through a 0.22 μm sterile pyrogen free filter and, further, heating at 100 °C for 30 min [20]. Later the endotoxic activity of the preparation is quantified by the use of Limulus amoebocyte lysate (LAL) assay [20,21,43,44].

The absence of a comprehensive ruminal LPS extraction protocol limits animal scientists to use, exclusively, *E. coli* LPS to investigate the inflammatory responses from ruminal acidosis in in vitro experiments of the rumen tissues [45,46]. Indeed, the presence of *E. coli* has been reported in the rumen under severe grain-induced SARA [47], and *E. coli* genes, that are potent virulence factors, have been identified in the rumen [48]. Despite that *E. coli* LPS virulence is well elucidated, the most predominant phylum in the rumen fluid is *Bacteroidetes*, even under SARA conditions [47,49]. *Bacteroidetes* LPS exhibit lower virulence than *E. coli* LPS [50,51]; however, we should not ignore the fact that ruminal LPS seems to be composed of an accumulation of LPS derived from different Gram-negative bacteria in the rumen, and therefore, LPS challenges the sole use of *E. coli* LPS, raising concerns regarding the representativeness of the results.

Overall, in order to ensure precision and reliability in experiments using ruminal LPS either for quantification or for in vitro challenges, the development of a complete standardized protocol for extraction, purification and analysis of ruminal LPS adapted to specificities of ruminal bacteria is of high importance.

## 3. Ruminal Lipopolysaccharides’ Immunogenicity and Biosynthesis

**Figure 1:**
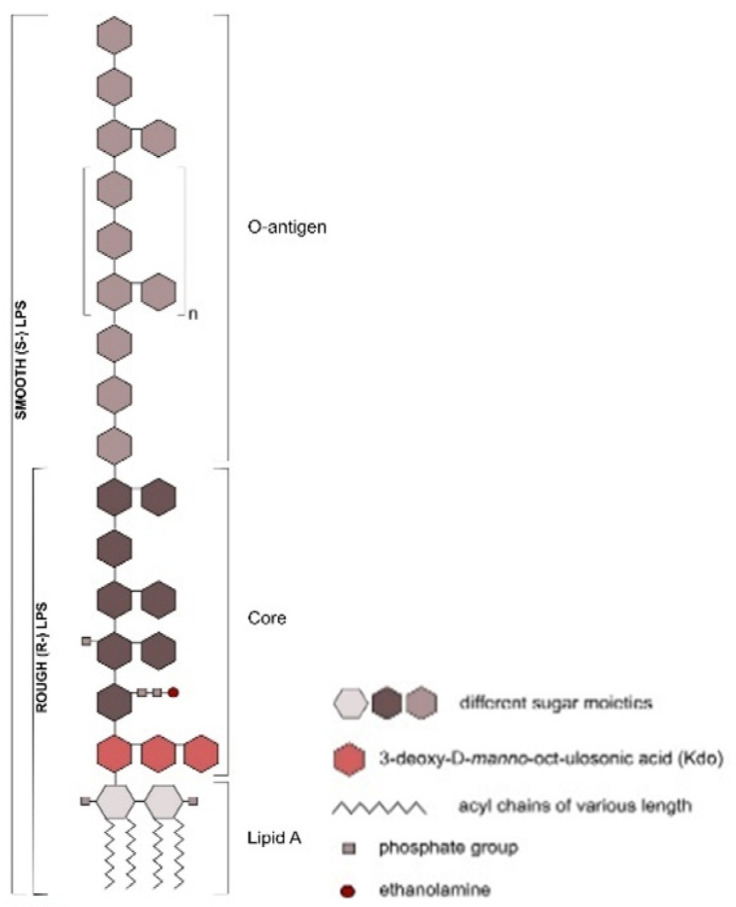
Diagram of the general structure of lipopolysaccharides of Gram-negative bacteria. Lipopolysaccharides of Gram-negative bacteria consist of three main subunits (from bottom to top): lipid A, the core region, and the O-antigen. Lipid A and the core (Adapted from Steimle, A. et al. [66]).

Lipopolysaccharide is an immunodominant molecule critical for the virulence and pathogenesis of many Gram-negative bacterial species, including *Salmonella spp*., *E. coli* and *Pseudomonas aeruginosa* [52,53,54], and differences in LPS O-antigen composition constitutes the basis for strains serotyping [55]. The LPS is composed of a large glycolipid tripartite molecule divided into three parts: a lipid A moiety, incorporated in the outer leaflet of the outer membrane of Gram-negative bacteria, a core oligosaccharide and repeating O-antigen units extended outward from the surface of the cell [55] (Figure 1). The LPS carbohydrate composition in the core region is important for bacterial membrane integrity and viability; specifically, heptose, when deleted, has been reported to be lethal in several virulence strains [56]. Thus, LPS monosaccharide composition analysis with gas chromatography mass spectrometry (GC-MS) could reveal key monosaccharides (e.g., heptose) that future research could target in strategies against SARA.

**Figure 2:**
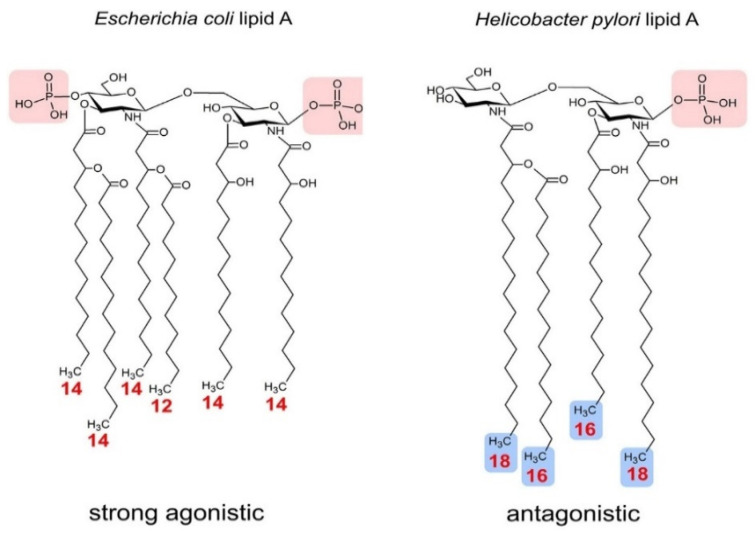
Structures of *Escherichia coli* (strong agonistic) and *Helicobacter pylori* (antagonistic). *Escherichia coli* Lipid A consists of six acyl chains (hexa-acylated) and two immunogenic phosphate groups (Light red); in contrast, *H. pylori* Lipid A is composed of four acyl chains (tetra-acylated) and one immunogenic phosphate group (light red). Structural differences of the acyl chains and modifications in phosphate groups significantly alter the immunogenic activity of Lipid A molecules (Adapted from Steimle, A. et al. [66]).

Besides the core region, another important moiety of LPS molecules is the lipid A. The number of acyl chains that a lipid A moiety consists of is usually correlated with the ability to induce cytokine production [57]. Therefore, possible variations of acyl chain numbers could potentially alter the host immune response whereas the hexa-acylated forms are usually considered as strong immunostimulant molecules [58] (Table 1). Furthermore, the number of phosphate groups on the lipid A portion make a critical contribution to its immunogenicity. In general, most lipid A structures consist of two phosphate groups (di-phosphorylated) but minor modifications or deletion of even one could turn an agonistic-lipid A into antagonistic-lipid A [59] (Figure 2).

*Escherichia coli* LPS is one of the most potent mediators of the inflammatory response in humans due to the expression of hexa-acylated, di-phosphorylated LPS [60]. In general, a hexa-acylation pattern indicates agonistic LPS and immunodominant bacteria species similar to *E. coli* [1,61] (Figure 2). The biosynthetic pathway of hexa-acylated lipid A from *E. coli* has been well elucidated by Raetz and Whitfield and its general mechanisms seems to be shared among most Gram-negative bacteria [62].

Contrary to the hexa-acylated lipid A from *E. coli*, *Prevotella spp* isolated from humans has been reported to produce penta-acylated lipid A structures, which is consistent with their low toxicity LPS [63]. In the rumen, *Prevotella* genus population has been reported to have no effect [64] or no increase in response to SARA induction [65]; however, the reason for this inconsistency remains unknown. Therefore, the characterization of the lipid A structure of *Prevotella* species in the rumen by using matrix-assisted laser desorption ionization time-of-flight mass spectrometry (MALDI TOF MS) could shed light on the potential of each specific specie to influence the innate immunity and enlighten any potential correlation with the host immune response triggered under SARA conditions.

### Ruminal Lipopolysaccharides’ Rough and Smooth Phenotypes

Lipopolysaccharide molecules are divided into two categories: (1) rough LPS or lipo-oligosaccharides (R-LPS), which is LPS with only lipid A and a core oligosaccharide component, and (2) smooth LPS (S-LPS), which is LPS capped with O-antigen [71]. The lipid A moiety is covalently linked to the inner core and carries the endotoxic activity of the LPS molecule [9,72].

Rough *E. coli* LPS are a challenge to mammary epithelial cells (MEC), indicating the crucial role of endotoxin receptor CD14 in the recognition of S-LPS, as well as revealing the pro-inflammatory response of MEC to LPS, modulated by the O-antigen [73]. In addition, the longest O-antigen polysaccharide chain has been reported to affect the secretion of cytokines in bovine blood neutrophils [74]. These results provide fundamental knowledge for future research in the development of novel diagnostic methods and/or the design of therapeutics against mastitis in dairy cows. However, *E. coli* LPS is associated with mastitis in dairy cows and it is available on the market, contrary to ruminal LPS for which there is no official extraction protocol, neither is it distributed by a company. Therefore, the absence of an extraction protocol for rough-LPS is a critical obstacle to researchers pursuing LPS challenges to rumen epithelial cells and tissues in order to expand their knowledge regarding SARA.

## 4. Lipopolysaccharide Sensing

Microbial-associated molecular patterns (MAMP) are recognized by germline-encoded receptors, called pattern recognition receptors (PRR), on the host cell membrane. One important family of PRR is that of the toll-like receptors (TLR). The TLR family has about 13 members which cover the recognition of the whole range of pathogens in vertebrates [75]. Toll-like receptor 4 (TLR4) has received notable attention from researchers because it is the signaling unit of the lipopolysaccharide-receptor complex [76]. Besides TLR4, there are three other glycoproteins involved in the binding, recognition, and response to the LPS. These are: (1) the CD14 molecule, either as membrane bound to TLR4-expressing cells [77] or as a soluble form [78]; (2) the lipid A binding protein (LBP), an acute phase protein, that removes LPS from the cell wall of bacteria [79,80]; (3) the small secreted molecule MD2 [81].

Lipopolysaccharides cannot be recognized by the host immune system, unless removed from the bacterial outer membrane, either by bacterial lysis or by the host LBP transferase [79]. The LBP transports LPS molecules to CD14 and forms the CD14–LPS complex [82]. Then, LPS is delivered to the ectodomain of the TLR4/MD2 receptor complex and, in hexa-acylated LPS, five out of six lipid chains are buried inside the hydrophobic pocket of MD2 and the other interacts with a second TLR4 molecule and leads to dimerization [83].

However, the dimerization of TLR4/MD2 complex is not always stimulated and has been reported to be dependent on LPS structure. More specifically, LPS from *E. coli* and *S. minnesota* cause the formation of dimeric TLR4 complexes, whereas LPS from *Rhodobacter sphaeroides*, an antagonistic chemotype, maintain monomeric TLR4 form in human embryonic kidney cells [84]. In rumen epithelial tissue, the ruminal LPS induce an inflammatory response through the NF- kB pathway during SARA [43]; however, how ruminal LPS binds to LBP, CD14 and MD2 has not yet been investigated. Importantly, by pursuing single molecule localization microscopy (SMLM) in the most abundant species of the bovine rumen microbiome under SARA conditions, a potential correlation between monomeric TRL4 and the antagonistic penta-acylated LPS from the abundant phylum of the rumen *Bacteroidetes* could be revealed.

## 5. Conclusions

Overall, the information presented in this review further substantiates the hypothesis that ruminal bacteria express under-acylated lipid A rather than hexa-acylated lipid A, which is how host-microbiome interactions are currently experimentally studied. In addition, it shows the importance of developing a comprehensive extraction, purification and analysis of ruminal LPS protocol to enlighten fundamental aspects of SARA and provide the basis for designing new strategies to understand and tackle ruminal acidosis. More specifically, the elucidation of the mechanisms that support host microbiome tolerance would be fundamental for the development of therapeutics for bovine nutrition disorders. Critical steps to achieve this would include the composition analysis of ruminal LPS sugars and fatty acids by GC MS, as well as the composition analysis of different acylation patterns exhibited from endotoxin derived from ruminal Gram-negative bacteria by MALDI TOF MS.

## Figures and Tables

**Table 1:** Acylation patterns of Lipid A moieties from different bacteria. The greater the number of acyl chains indicates stronger immunogenicity of the respective lipopolysaccharide (LPS) molecule.

Bacteria Species LPS	Acylation Pattern	Biological Properties			Reference
		Human	Hamster	Mouse	
*E. coli* O55:B5	Hexa-acylation	Strong Agonist	Strong Agonist	Strong Agonist	[67]
*Porphyromonas gingivalis* (Periodontopathogen)	Penta-or Tetra-acylation	Weak Agonist- Antagonist	-	-	[68]
*Helicobacter pylori*	Tetra-acylation	Weak Agonist	-	-	[69]
*Salmonella minnesota* (Di-phosphoryl-lipid A)	Hexa-acylation	Agonist	-	Agonist	[70]
